# Association between Stress Response Genes and Features of Diurnal Cortisol Curves in the Multi-Ethnic Study of Atherosclerosis: A New Multi-Phenotype Approach for Gene-Based Association Tests

**DOI:** 10.1371/journal.pone.0126637

**Published:** 2015-05-20

**Authors:** Zihuai He, Erin K. Payne, Bhramar Mukherjee, Seunggeun Lee, Jennifer A. Smith, Erin B. Ware, Brisa N. Sánchez, Teresa E. Seeman, Sharon L. R. Kardia, Ana V. Diez Roux

**Affiliations:** 1 Department of Biostatistics, University of Michigan, Ann Arbor, United States of America; 2 Life Sciences Program, Northrop Grumman Health Division, McLean, Virginia, United States of America; 3 Department of Epidemiology, University of Michigan, Ann Arbor, United States of America; 4 Department of Medicine, Division of Geriatrics, David Geffen School of Medicine, University of California Los Angeles, Los Angeles, United States of America; 5 Department of Epidemiology, Drexel University, Philadelphia, United States of America; University of California Irvine, UNITED STATES

## Abstract

The hormone cortisol is likely to be a key mediator of the stress response that influences multiple physiologic systems that are involved in common chronic disease, including the cardiovascular system, the immune system, and metabolism. In this paper, a candidate gene approach was used to investigate genetic contributions to variability in multiple correlated features of the daily cortisol profile in a sample of European Americans, African Americans, and Hispanic Americans from the Multi-Ethnic Study of Atherosclerosis (MESA). We proposed and applied a new gene-level multiple-phenotype analysis and carried out a meta-analysis to combine the ethnicity specific results. This new analysis, instead of a more routine single marker-single phenotype approach identified a significant association between one gene (ADRB2) and cortisol features (meta-analysis p-value=0.0025), which was not identified by three other commonly used existing analytic strategies: 1. Single marker association tests involving each single cortisol feature separately; 2. Single marker association tests jointly testing for multiple cortisol features; 3. Gene-level association tests separately carried out for each single cortisol feature. The analytic strategies presented consider different hypotheses regarding genotype-phenotype association and imply different costs of multiple testing. The proposed gene-level analysis integrating multiple cortisol features across multiple ethnic groups provides new insights into the gene-cortisol association.

## Introduction

Cortisol concentrations follow a strong daily pattern. They are high upon awakening, reach a maximum concentration approximately half an hour later, and slowly decrease throughout the rest of the day [1]. Additionally, cortisol concentrations increase in response to stressful situations, such as public speaking [2]. Under conditions of chronic stress, prolonged increased concentrations could have detrimental downstream physiological effects. Several population-based studies have linked daily cortisol patterns to health outcomes, including elevated blood pressure, abdominal obesity, and coronary calcification [3–5]. Cortisol concentrations and various features of the cortisol daily profile have also been linked to diabetes mellitus [6] and markers of inflammation [7]. Despite evidence of associations of various risk factors with cortisol, considerable inter-individual variability in cortisol remains unexplained. This has led to increased interest in examining genetic predictors of cortisol phenotypes. Most genetic research on cortisol to date has focused on candidate gene associations, notably the glucocorticoid receptor gene (NR3C1) and the mineralocorticoid receptor gene (NR3C2) [8–9].

The increase in cortisol concentrations in response to a stressor occurs through the activation of the hypothalamic-pituitary-adrenal (HPA) axis, which has effects on the cardiovascular system, the immune system, and metabolism [10]. The cortisol metabolic pathway suggests several key genes whose variation could affect cortisol levels. A number of genes are likely involved in the physiologic responses to stress. We selected genes known to play a role in stress reactivity and/or in responses to stress. The physiological roles of these genes were deduced from a large body of research in molecular biology, animal models, and a variety of human populations. This was done solely based on existing literature. MESA data was not used in any manner to generate the list of candidate genes. The six genes of interest for this work include the glucocorticoid receptor gene (NR3C1) and the mineralocorticoid receptor gene (NR3C2) (which act in concert to regulate cortisol responses to psychological stressors [11]), the tyrosine hydroxylase gene (TH), the alpha-2A-adrenergic receptor gene (ADRA2A), the beta-2-adrenergic receptor gene (ADRB2) (all three of which are related to cateholamine synthesis and reactivity to stress [12–15]), and the serotonin transporter gene (SLC6A4) (which may modulate various responses to stress [16]). These six genes appear to be involved in the response to psychological stressors [11, 13–16] and could therefore impact cortisol levels. However, few if any population-based studies have investigated the associations between polymorphisms in these genes and cortisol levels in multiple ethnic groups.

In this study we investigate how variation in these six genes is related to seven diurnal cortisol features within and across ethnic groups. In an attempt to better characterize the biological processes involved, the seven cortisol features are defined as mathematical functions of measurements collected six times per day over three consecutive weekdays. This is an advance over prior work that has focused on single measurements of cortisol, which are likely to be imperfect markers of the real phenotype of interest (the actual shape and level of the entire cortisol curve) [17]. Moreover, jointly modeling these cortisol features can additionally improve power over a single-feature analysis, as it allows identification of genetic associations that are present in multiple phenotypes. Several methods for analyzing multiple phenotypes jointly for single marker tests have been proposed recently [18–20].

Recent studies showed the advantages of gene-level analysis over individual single nucleotide polymorphism (SNP) analyses [21, 22]. When comparing SNP-level results across multiple ethnic groups, differences in ethnicity specific linkage disequilibrium structures may result in inconsistent findings. Since gene structure (exon and intron organization) is not likely to differ across ethnic groups, making the assessment of entire genes is likely to be a better analytic approach than analyzing individual SNPs in a multi-ethnic cohort. The gene-level analysis bypasses the problem that different tagging SNPs within gene regions may index relevant variation across ethnic groups, and reduces the burden of multiple testing. However, there is no published method so far that can handle gene-based inference with multiple correlated phenotypes.

We contrasted and compared four different analytic strategies with different hypotheses, all targeted towards gene discovery, resulting in varying number of tests: 1. Standard strategy which tests association of single SNPs (approximately 500 markers located on the six candidate genes), one at a time with each cortisol feature (~500 x 7 = 3500 tests in our study); 2. Multivariate association tests of single SNPs with multiple cortisol features (~ 500 x 1 = 500 tests); 3. Gene-level (defined as all SNPs within the gene and a 5 kilobases (kb) window up- and downstream of each gene) SNP-set analysis for a single cortisol feature (6 x 7 = 42 tests); 4. A new gene-level multiple-phenotype analysis (only 6 tests) we proposed for summarizing the gene-level results of the seven cortisol features. In each approach we test for different hypotheses, for example, marginal association of a single SNP with a single outcome, joint association of multiple outcomes with a SNP, joint association of a SNP-set with a single outcome, joint association of a SNP-set with multiple outcomes. Bonferroni correction applied to any of these set of tests controls for the family-wise error rate or the probability of rejecting at least one true null hypothesis when the global null hypotheses of no association holds. However, one has to remember that the family of tests being performed in each method and the inferential focus as stated in terms of the respective null hypotheses is different, that in turn, leads to different number of tests and different cost of multiple testing. For each analysis we carried out meta-analysis to combine the ethnicity specific results. Among the six stress response genes investigated, the new gene-level multiple-phenotype analysis identified a significant association between one gene (ADRB2) and cortisol features (meta-analysis p-value = 0.0025), while none of the meta-analysis results of the other three strategies are significant after Bonferroni correction.

## Materials and Methods

### Study Participants

We analyzed data from the MESA Stress Study, which is an ancillary study to the original MESA study, a longitudinal cohort study focused on investigating the early stages of atherosclerosis. The MESA Stress Study was approved by the Institutional Review Boards at UCLA and Columbia University. This research was also approved by the Health Sciences and Behavioral Sciences Institutional Review Board at the University of Michigan. The data was anonymized and de-identified prior to analysis. All procedures were carried out with the adequate understanding and written consent of the subjects. Eligible participants were 45–84 years of age and free from history of cardiovascular disease at the baseline examination (2000–2002) [23]. The MESA Stress Study took place in the context of MESA examinations 3 and 4 conducted between 2004 and 2006, and obtained detailed stress hormone data on a subsample of 1002 MESA participants recruited from the New York and Los Angeles sites. Participants for the MESA Stress Study were African Americans, European Americans, and Hispanic Americans and were enrolled as they presented for follow-up, until approximately 500 participants were recruited from each location. Of the 1002 MESA Stress Study participants, after exclusions for 1) no consent for use of genetic information, and 2) quality control processes, our resultant sample size was 950 individuals. The ethnicity specific distribution of this sample is as follows: 181 European Americans, 254 African Americans, and 515 Hispanic Americans. Table 1 presents more descriptive statistics of the study.

**Table 1:** Characteristics of MESA Stress Study participants.

Characteristics	EUR (N = 181)	AFA (N = 254)	HIS (N = 515)	ALL (N = 950)
Age				
45–54	28 (15.5%)	42 (16.5%)	88 (17.1%)	158 (16.6%)
55–64	50 (27.6%)	84 (33.1%)	161 (31.3%)	295 (31.1%)
65–74	56 (30.9%)	82 (32.3%)	177 (34.3%)	315 (33.2%)
75 +	47 (26.0%)	46 (18.1%)	89 (17.3%)	182 (19.1%)
Gender				
Female	93 (51.4%)	138 (54.3%)	263 (51.1%)	494 (52.0%)
Male	88 (48.6%)	116 (45.7%)	252 (48.9%)	456 (48.0%)
Site				
New York	101 (55.8%)	158 (62.2%)	214 (41.6%)	473 (49.8%)
Los Angeles	80 (44.2%)	96 (37.8%)	301 (58.4%)	477 (50.2%)
Education				
High school or less	9 (5.0%)	23 (9.1%)	230 (44.7%)	262 (27.6%)
Completed high school	23 (12.7%)	59 (23.2%)	112 (21.7%)	194 (20.4%)
Some college	41 (22.6%)	115 (45.3%)	124 (24.1%)	280 (29.5%)
Bachelor’s or higher	108 (59.7%)	57 (22.4%)	49 (9.5%)	214 (22.5%)
Cortisol Features	Mean (SD)			
Wakeup	2.57 (0.55)	2.36 (0.59)	2.34 (0.62)	2.39 (0.60)
Cortisol awakening	0.44 (0.47)	0.33 (0.47)	0.39 (0.53)	0.38 (0.50)
Bedtime	0.77 (0.79)	0.94 (0.75)	0.47 (0.83)	0.65 (0.83)
Area under the curve	1.64 (0.43)	1.57 (0.46)	1.44 (0.51)	1.52 (0.49)
Early decline slope	-0.53 (0.38)	-0.43 (0.44)	-0.39 (0.44)	-0.43 (0.43)
Overall decline slope	-0.11 (0.07)	-0.10 (0.05)	-0.12 (0.06)	-0.11 (0.06)
Late decline slope	-0.11 (0.06)	-0.10 (0.07)	-0.13 (0.06)	-0.12 (0.07)

EUR: European Americans. AFA: African Americans. HIS: Hispanic Americans. SD: standard deviation. Cortisol concentrations (nmol/L) were log-transformed and averaged across the three days of collection to create each feature.

### Cortisol Features

Each MESA Stress Study participant was asked to collect six saliva samples per day at pre-specified times over three consecutive weekdays, for a maximum of 18 samples, using Salivette collection tubes. The samples were collected using the following schedule: Sample 1) upon waking and before getting out of bed; 2) 30 minutes later; 3) Around 10:00am; 4) Around 12:00 noon or before lunch, whichever came first; 5) Around 6:00pm or before dinner, whichever came first; 6) just before bed. Because earlier work has shown that the use of a time tracking device improves sample collection compliance [24], each collection tube was equipped with a time tracking device, which recorded the time when the swabs were removed for sample collection.

**Fig 1:**
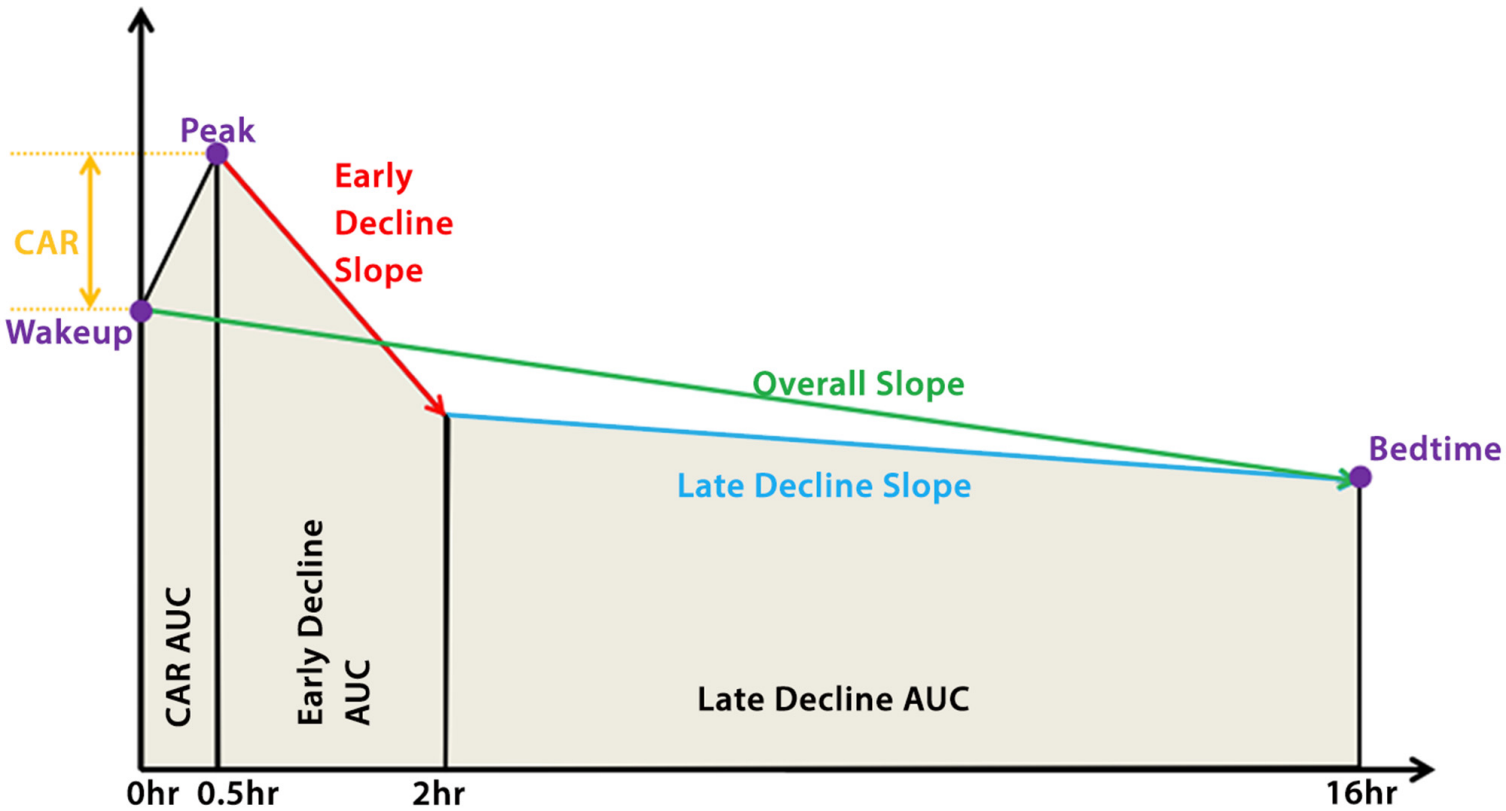
Representation of the diurnal cortisol curve describing our summary features of interest. In our study we specifically used Wakeup, Bedtime, Cortisol awakening response (CAR), Area under the curve (AUC) from 0–16 hours, Early Decline Slope, Late Decline Slope, and Overall Decline Slope.

We explored seven features of the diurnal cortisol cycle averaged across the three days for each individual (Fig 1, Table A in S1 Table): 1. Cortisol concentration at wakeup (Wakeup); 2. Difference in cortisol concentrations between the peak and wakeup measurements (CAR); 3. The slope from 0.5 hours and 2 hours since wakeup (EDSlope); 4. The slope from 2 hours to 16 hours since wakeup (LDSlope); 5. The overall decline slope ignoring the peak value from wakeup to bedtime pooled (ODSlope); 6. The cortisol concentration at bedtime (Bedtime); 7. Standardized area under the curve for the interval 0hr-16hr since wakeup averaged across all days for an individual (AUC). Features were selected for investigation because prior work has hypothesized or demonstrated their associations with health risk factors or health outcomes [6, 7, 25, 26]. Features were modeled using all available salivary cortisol data (up to six samples per day collected over three days). Raw cortisol concentrations, measured in nmol/L, were log-transformed to more closely approximate a normal distribution [25]. Descriptive statistics can be found in Table 1. Correlation across multiple cortisol features were calculated and summarized in Table B (in S1 Table).

### Genetic Information

Genotyping data included both measured and imputed SNPs available through participation in the MESA SHARe (SNP Health Association Resource) project. Under the SHARe project, genome-wide genotyping was obtained using the Affymetrix Genome-Wide Human SNP Array 6.0 platform. Imputation to HapMap was completed at the MESA Genetics Centers using the IMPUTE2 [27] program with the following reference panels: the HapMap Phase I and II, the human genome reference sequence (NCBI Build 36) [28]. Imputation for African Americans and Hispanic Americans was performed using the CEU+YRI+CHB+JPT reference panels (release #22). Imputation for European Americans was performed using only the CEU reference panel (release #24). Based on the imputed allele probabilities (AA, AB, BB), dosage data was created and used for the analysis.

We defined the six stress response gene regions as the entire gene, plus a window ±5kb around each gene. Base pair start and end positions for each gene were assigned based on annotation from the UCSC Genome Browser [29], and the analysis was restricted to common SNPs with minor allele frequency (MAF) >5%. Due to the small ethnic group specific sample sizes, a threshold for the MAF of 5% was chosen to limit the influence of unstable frequency estimates being driven by small sample sizes. Details on the chromosomal locations of each of the six genes, overall size, and the number of SNPs in each gene region by ethnic group can be found in Table 2.

**Table 2:** Characteristics of the stress response genes.

Region Name	Full Name	Chromosomal Location	Start (bp)	End (bp)	Size (bp)	Number of SNPs		
						EUR	AFA	HIS
ADRA2A	Alpha-2A-adrenergic receptor gene	10q24-q26	112,826,911	112,830,560	3650	9	12	11
ADRB2	Beta-2-adrenergic receptor gene	5q31-q32	148,186,349	148,188,381	2,033	16	18	18
NR3C1	Glucocorticoid receptor gene	5q31.3	142,637,689	142,795,270	157,582	73	82	71
NR3C2	Mineralocorticoid receptor gene	4q31.1	149,219,365	149,583,093	363,729	322	361	322
SLC6A4	Serotonin transporter gene	17q11.1-q12	25,549,032	25,586,841	37,810	25	32	26
TH	Tyrosine hydroxylase gene	11p5.5	2,141,735	2,149,611	7,877	15	16	15

Start/End position is according to the smallest start / largest end of the gene by UCSC genome browser based on the March 2006 human reference sequence (NCBI Build 36.1) produced by the International Human Genome Sequencing Consortium. Genotype data used for the main analysis includes both measured and imputed common SNPs (minor allele frequency > 0.05) in each gene +- 5kb. The imputation is based on ethnic specific reference panels. EUR: European Americans. AFA: African Americans. HIS: Hispanic Americans.

### Statistical Analysis

We conducted ethnicity specific analysis suggested by the evaluation of inter-ethnic variability of cortisol features using ANOVA by which race has an association with cortisol features (Table C in S1 Table). We adjusted for age and sex as precision variables related to the outcome (cortisol features), consistent with previous MESA publications [30, 31, 32]. Since socio-economic status was previously shown to be associated with cortisol levels in MESA [33], we also examined study site and education as possible precision variables (these can be thought of as a proxy for socio-economic status), but found no evidence of a strong association with cortisol features. Thus these two predictors were not included in the model. We included top five ethnicity specific genetic principal components as potential confounders to account for racial differences not captured by self-reported race (in the full sample) or genetic admixture (within each ethnic group). Bonferroni correction was applied for multiple comparison adjustment in all analytic strategies introduced below.

#### Method 1. Single SNP Analysis for single cortisol feature

For each ethnic group, we applied multivariable linear regression to investigate associations between each cortisol feature and each SNP separately. Given the seven cortisol features and approximately 500 SNPs in total (460 in European Americans, 526 in African Americans and 463 in Hispanic Americans), this strategy results in 7×500 = 3500 tests. The specific model is as follows:

$$Y_{ik}=\alpha_{0k}+\alpha_{k}^{'}X_{i}+\beta_{k}g_{i}+\varepsilon_{ik},$$

where *Y*_*ik*_ is the *k*-th cortisol feature *(k = 1*,…,*K* in general with *K =* 7 for this example*)* corresponding to subject *i*; *α*_0*k*_ is an intercept term; **X**_**i**_ is a vector of non-genetic covariates; *g*_*i*_ is a minor allele count of single SNP in the regions (*g*_*i*_ = 0,1,2) and measurement error *ε*_*ik*_ follows any distribution with mean zero and variance $\sigma_{k}^{2}$. ***α***_*k*_ is a vector of regression coefficients for the covariates, and *β*_*k*_ is a regression coefficient for the SNP regressed on the *k*-th cortisol feature. Standard Wald test is used to test the null hypothesis *H*_0_:*β*_*k*_ = 0.

#### Method 2. Single SNP analysis with joint test for multiple cortisol features

We then applied the MultiPhen method to test for the joint association of the multiple cortisol phenotypes with a given SNP [18]. This strategy results in approximately 500 tests (approximate number of SNPs). MultiPhen inverts the regression problems so that the SNP genotypes become the outcome variable, and the phenotypes become the covariates in a proportional odds logistic regression. The model is given by:

$$P(g_{i}\leq m)=\frac{1}{1+e^{-\alpha_{0gm}-\alpha_{gm}^{'}X_{i}-\sum_{k=1}^{K}\beta_{gk}Y_{ik}}}$$

where *m* (*m* = 0,1,2) is a minor allele count of SNP*g*_*i*_; *Y*_*ik*_ is the *k*-th cortisol feature corresponding to subject *i*; *α*_0*gm*_ is an intercept term; **X**_**i**_ is a vector of non-genetic covariates. ***α***_***gm***_ is a vector of regression coefficients for the covariates, and *β*_*gk*_ is a regression coefficient for the *k*-th cortisol feature. Then it uses a likelihood ratio test with *K* degrees of freedom to test the global null hypothesis *H*0:*β*_*g*1_ = … = *β*_*gk*_ = 0. Since MultiPhen cannot be directly applied to dosage data, we use best call genotype data for this analysis.

Note that though MultiPhen can aggregate the information across multiple phenotypes, the statistical justification for covariate adjustment in such a reverse regression setting with the proportional odds link function remains unclear. The method only tests for conditional association of genes and outcomes (conditional on covariates) and is not designed to unbiasedly estimate the beta coefficients or arrive at a causal interpretation. We also note that MESA is a cohort study and modeling the genes as outcomes is not interpretable and the reverse regression setting is a mere statistical artifact to construct the multivariate tests for association. One cannot interpret the beta-coefficients obtained from this model of **G**|**Y**,**X** the method is only used to derive joint association test for the global null hypotheses that none of the multiple outcomes are related to the single genetic marker.

#### Method 3. Gene-level analysis for each cortisol feature separately

To assess the impact of the six stress response genes on cortisol features, for each ethnic group, we performed gene-level analysis using the sequence kernel association test (SKAT) separately for each pair of cortisol feature and gene region [22]. This strategy results in 7×6 = 42 tests. The model for testing genetic main effects is as follows:

$$Y_{ik}=\alpha_{0k}+\alpha_{k}^{'}X_{i}+\beta_{k}^{'}G_{i}+\varepsilon_{ik},$$

where *Y*_*ik*_ is the *k*-th cortisol feature corresponding to subject *i*; *α*_0*k*_ is an intercept term; **X**_**i**_ is a vector of non-genetic covariates; **G**_**i**_ a vector of genotypes and measurement error *ε*_*ik*_ follows any distribution with mean zero and variance $\sigma_{k}^{2}$. ***α***_*k*_ is a vector of regression coefficients for the covariates, and ***β***_*k*_ = (*β*_1*k*_,…,*β*_*pk*_)' is a vector of regression coefficients for the genotypes corresponding to the *p* markers on a gene. In SKAT, one assumes that each of the *β*_*jk*_, *j = 1*,*…*,*p*, independently follows an arbitrary distribution with mean zero and variance $w_{jk}\tau_{k}^{2}$, where *w*_*jk*_ is a weighting parameter to up-weight rarer variants. Testing $H_{0}:\tau_{k}^{2}=0$ is equivalent to testing *H*_0_:***β***_*k*_ = 0, i.e., *H*_0_:*β*_*jk*_ = 0 for *j = 1*,*…*,*p*. We used *w*_*jk*_ = 1 for all SNPs since the weighting scheme is developed for rare variant association tests and hence not necessary for testing for common variant associations. The asymptotic null distribution of the resultant score statistic is a mixture of chi-squares [22].

#### Method 4. Gene-level multiple-phenotype analysis for jointly testing 7 cortisol features

We proposed and applied a new multiple phenotype analysis to integrate the information contained in the seven cortisol features but still using a gene as the unit of analysis. This strategy results in only 6 tests (number of genes). The method appropriately combines p-values of the gene-level tests corresponding to each individual cortisol feature obtained in Method 3 and provides a combined p-value using Fisher’s combined probability test [34]. In order to account for the correlation among cortisol features, and thus the gene-level statistics, a permutation procedure was then followed to adjust the combined p-value across multiple features, described as follows:

Step 1: Randomly permute the subject ID linked to the genotype data to reflect the null hypothesis of no association between the cortisol features and gene regions.

Step 2: Apply SKAT (Method 3) separately for each pair of cortisol feature and gene region, adjusting for the confounders. The model with permuted genotype data is given by:

$$Y_{ik}=\alpha_{0k}+\alpha_{k}^{'}X_{i}+\beta_{k}^{'}G_{i*}+\varepsilon_{ik}$$

where **G**_i*_ is now a vector of the genotypes corresponding to another randomly chosen subject i*.

Step 3: Use Fisher’s combined probability test to combine the test for each cortisol feature and compute a combined p-value.

Step 4: Repeat Step 1–Step 3 for 10000 times and get the distribution of the combined p-values under the null hypothesis.

Step 5: Adjust the observed p-value and determine significance using the null distribution of the combined p-value generated by the permutation procedure.

Because the permutation only destroys the association between cortisol features and gene regions but retains the correlation structure among cortisol features, this procedure can account for the correlation structure of multiple phenotypes to take advantage of the rich information contained in the seven cortisol features and leading to a single multivariate association test P-value capturing gene-level association.

A limitation of the above permutation approach is that it cannot account for the possible correlation between genotypes and covariates. Genotype-covariate association may be expected, for example when principal components are used as covariates to adjust for population stratification. As a result, such a permutation procedure may not provide entirely accurate Type I error rates in some scenarios [35].

#### Meta Analysis

Given that we have a multi-ethnic sample and the association may vary across groups, we employed meta-analysis for both gene-level analysis and single SNP analysis using three different tests: 1. Weighted Z-score test [36]; 2. Fisher’s combined probability test; 3. Weighted Fisher’s combined probability test [37]. The tests allow for a meta-analysis of genetic effects across ethnic groups only using the ethnicity specific p-values.

## Results

Basic demographic and health status information on the 950 Stress Study participants at baseline is provided in Table 1. Hispanic Americans represented the largest proportion of participants (54.2%), relative to the African Americans (26.7%) and European Americans (19.1%). The gender distribution was fairly equal (52.0% female). Overall, cortisol feature means varied across ethnic groups. There was a statistically significant difference in means across the ethnic groups for all cortisol features except CAR (Table C in S1 Table). We ran ANOVA to assess the impact of the covariates on cortisol features. Age and gender were significant for multiple cortisol features across ethnic groups; Site was only significant for Wakeup and ODSlope in Hispanic Americans; Education did not show significant association (Table C in S1 Table). The correlation structure of seven cortisol features can be found in Table B (in S1 Table) and we can observe some strong correlations (maximum of 0.77).

The most significant SNP in each gene identified by the single-SNP analysis (Methods 1 and 2) are summarized in Table D (in S1 Table). We note that the single-SNP meta-analysis exhibits some small p-values, but none of these p-values is significant after Bonferroni correction (Bonferroni threshold = 1.4×10^−5^ for single cortisol analysis, 0.0001 for MultiPhen) due to the burden of multiple comparisons and the limited sample size (950 individuals after exclusions). These results are used as supportive information to further evaluate the association via other methods.

The results for the gene-level assessment of gene-by-cortisol associations by Method 3 are presented in Table 3. Three genes, ADRA2A, ADRB2 and NR3CA, exhibited small p-values (less than 0.05) with more than one cortisol features in at least one ethnic group, but none of the single cortisol by gene analysis p-values are significant after Bonferroni correction (Bonferroni threshold = 0.0012). We then further combined the p-values obtained by Method 3 for separate cortisol features and applied the permutation procedure in Method 4 to produce a single p-value combining all seven features. After Bonferroni correction for testing of the six stress response genes (Bonferroni threshold = 0.0083), only *ADRB2* exhibited significant association with cortisol features by the multi-phenotype meta-analysis using Fisher’s combined probability test (p-value = 0.0025). The association was most significant in European Americans (p-value = 0.0019) less significant in African Americans (p-value = 0.0342), and not significant in Hispanic Americans (p-value = 0.6073). We note that the p-values of the weighted tests are larger because the weighted tests assign a higher weight on Hispanic Americans. There were four instances where the association p-value was less than 0.05 among the cortisol features by meta-analysis: CAR (p-value = 0.0176), Bedtime (p-value = 0.0261), AUC (p-value = 0.0443) and ODSlope (p-value = 0.0262). The association pattern is not uniformly consistent among ethnic groups and cortisol features: *ADRB2* is associated with CAR, Bedtime and LDSlope in European Americans, but only associated with AUC and ODSlope in African Americans and not significant in Hispanic Americans.

**Table 3:** Gene based analysis testing the association between the cortisol features and common variants (minor allele frequency > 0.05) in the stress response genes using SKAT (Method 3).

	Race	Wakeup	CAR	Bedtime	AUC	EDSlope	ODSlope	LDSlope	MP-Fisher
ADRA2A	EUR	0.9583	0.2742	0.1461	0.0705	0.0906	0.7720	0.7337	0.2479
	AFA	0.1022	0.0605	0.9062	0.8741	0.6721	0.1247	0.8161	0.3206
	HIS	**0.0379**	0.3480	**0.0497**	0.0819	0.6038	0.1470	0.2229	0.0528
	Meta-WZ	0.0626	0.1212	0.1209	0.1251	0.5045	0.1231	0.4662	**0.0371**
	Meta-F	0.0826	0.1122	0.1227	0.1024	0.3588	0.2026	0.6729	0.0900
	Meta-WF	**0.0365**	0.1592	0.0715	0.0891	0.5182	0.1393	0.4549	0.0551
ADRB2	EUR	0.3137	**0.0042**	**0.0071**	0.2690	0.2433	0.0642	**0.0375**	**0.0019**
	AFA	0.0582	0.3100	0.1640	**0.0271**	0.3515	**0.0256**	0.9451	**0.0342**
	HIS	0.4696	0.3507	0.6614	0.2150	0.5480	0.4733	0.6374	0.6073
	Meta-WZ	0.1911	0.0920	0.2137	**0.0472**	0.3948	0.0906	0.6699	0.0799
	Meta-F	0.1464	**0.0176**	**0.0261**	**0.0443**	0.4098	**0.0262**	0.2704	**0.0025**
	Meta-WF	0.2160	0.0852	0.1381	0.0680	0.4910	0.0873	0.4839	**0.0382**
NR3C1	EUR	0.1435	0.9246	0.4471	0.3017	0.7611	0.2246	0.9799	0.5870
	AFA	0.5316	0.1734	0.1237	0.2981	0.6650	0.9120	0.5822	0.4638
	HIS	0.0958	0.9425	0.3305	0.1115	0.2529	**0.0308**	**0.0445**	**0.0482**
	Meta-WZ	0.0802	0.9167	0.1832	0.0775	0.4301	0.1046	0.2263	0.0816
	Meta-F	0.1317	0.7065	0.2378	0.1624	0.6616	0.1192	0.2898	0.1932
	Meta-WF	0.1041	0.7730	0.2466	0.1176	0.4690	0.0521	0.0994	0.0810
NR3C2	EUR	0.3648	0.2545	0.3486	0.4280	0.9595	0.1706	0.2389	0.3628
	AFA	0.7560	0.7089	0.6848	0.8687	0.9077	0.8365	0.5606	0.9838
	HIS	0.9484	0.7422	0.6649	0.5489	0.9915	0.3854	0.7366	0.9455
	Meta-WZ	0.9433	0.7223	0.6737	0.6995	0.9991	0.4515	0.6526	0.9849
	Meta-F	0.8476	0.6738	0.7197	0.7861	0.9995	0.4459	0.5918	0.9032
	Meta-WF	0.9276	0.7739	0.7667	0.7657	0.9997	0.4724	0.7100	0.9607
SLC6A4	EUR	0.5724	**0.0392**	0.4734	0.3926	0.1955	0.5366	0.3807	0.2551
	AFA	0.4337	0.7558	0.8128	0.2055	0.3346	0.0943	0.3360	0.3768
	HIS	0.5460	0.9706	0.8793	0.8972	0.0601	0.8535	0.3693	0.7063
	Meta-WZ	0.5331	0.9161	0.9126	0.7434	**0.0386**	0.6452	0.2893	0.5532
	Meta-F	0.6770	0.3120	0.9037	0.5120	0.0860	0.3922	0.4115	0.4961
	Meta-WF	0.6575	0.6171	0.9424	0.6557	0.0588	0.5192	0.3979	0.6158
TH	EUR	**0.0499**	0.1964	0.3484	0.1867	0.3554	0.2974	0.5254	0.1458
	AFA	0.7118	0.2384	0.4130	0.3802	0.1127	0.6316	0.0982	0.2628
	HIS	0.5535	0.5448	0.0591	0.0990	0.4491	0.1065	0.0861	0.0916
	Meta-WZ	0.4427	0.3224	0.0610	0.0671	0.2320	0.1394	**0.0452**	**0.0424**
	Meta-F	0.2485	0.2908	0.1456	0.1282	0.2356	0.2514	0.0936	0.0794
	Meta-WF	0.4184	0.3936	0.0781	0.1001	0.2900	0.1595	0.0651	0.0715

MP-Fisher in the last column is the proposed multi-phenotype analysis (Method 4) that combines the gene-based p-values across the seven cortisol features.

CAR: cortisol awakening response. AUC: area under the diurnal cortisol curve. EDSlope: early decline slope. ODSlope: overall decline slope. LDSlope: Late decline slope. MP-Fisher: The Fisher’s probability test combining the seven cortisol features where permutation is used to account for the correlation among cortisol features. EUR: European Americans. AFA: African Americans. HIS: Hispanic Americans. Meta-WZ [36]: meta-analysis using weighted Z-score test. Meta-F: meta-analysis using Fisher’s probability test. Meta-WF [37]: meta-analysis using weighted Fisher’s probability test. Each cell presents the p-value. Age, gender and top five principal components were adjusted as covariates. Each cell presents the p-value. P-values less than 0.05 are bolded. Gene-level Bonferroni threshold is 0.0012 for single cortisol feature analysis, and 0.0083 for multiple cortisol features analysis.

**Fig 2:**
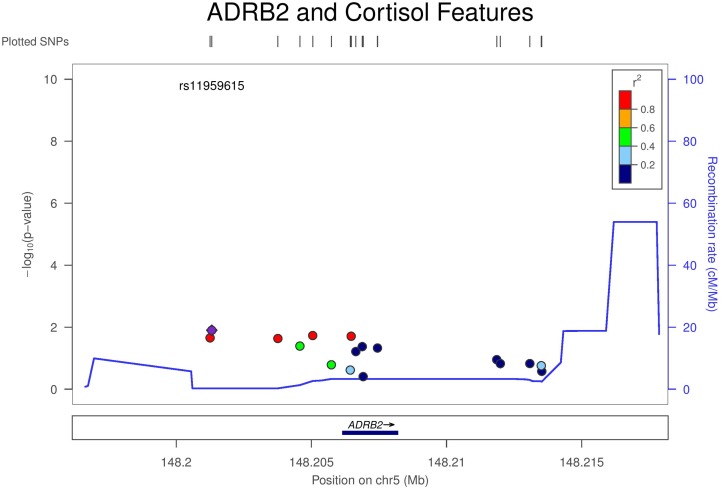
LocusZoom plot of the association between all SNPs in the *ADRB2* gene region and cortisol features. Each SNP in the *ADRB2* gene region was analyzed by MultiPhen (O’Reilly et al., 2012) stratified by ethnicity. Then the meta-analysis p-values were calculated using Fisher’s probability test and plotted using LocusZoom (Pruim et al., 2010). The linkage disequilibrium is based on the European Americans. This is labeled as Method 2 in the paper.

To further investigate the association between *ADRB2* and multiple cortisol features at a SNP level, we examined in more detail the individual SNP-by-cortisol joint association in this region as produced by Method 2. Results for this association using meta-analysis of MultiPhen p-values are shown using LocusZoom in Fig 2 [38]. The linkage disequilibrium structure was based on European Americans which exhibited the most significant signal. SNP level results stratified by ethnic groups can be found in S1–S3 Figs. The most strongly associated SNP is rs6580583 (meta-analysis p-value using Fisher’s method = 0.0051) which had the small p-values in European Americans (p-value = 0.0121) and Hispanic Americans (p-value = 0.0140) but was not significant in African Americans (Table 4). The other two suggestive SNP findings in ADRB2 are rs17778257 and rs12654778 (meta-analysis p-value using Fisher’s method = 0.0091 and 0.0072 respectively). From the present analysis we can observe that gene-level analysis is more consistent than single SNP analysis across ethnic groups and leveraging correlation among multiple cortisol features, in turn, reducing the number of tests can aid with enhancing the power of such an analysis.

**Table 4:** P-values for the top SNP in ADRB2 gene associated with the seven cortisol features according to multi-phenotype meta analysis (Method 2).

SNP Name	Race	Wakeup	CAR	Bedtime	AUC	EDSlope	ODSlope	LDSlope	MultiPhen
rs6580583	EUR	**0.0471**	0.7748	**0.0017**	0.0645	**0.0352**	0.1067	**0.0087**	**0.0121**
	AFA	0.8686	0.6376	0.1961	**0.0193**	0.0741	0.1071	0.8007	0.5619
	HIS	0.8717	0.9169	0.4227	0.4052	0.5622	0.4950	0.8090	**0.0140**
	Meta-WZ	0.8263	0.9406	0.0795	0.0625	0.1539	0.1817	0.6513	**0.0059**
	Meta-F	0.3527	0.9537	**0.0068**	**0.0188**	**0.0422**	0.1106	0.1100	**0.0051**
	Meta-WF	0.6281	0.9655	0.0600	0.0657	0.1392	0.2111	0.3610	**0.0066**

CAR: cortisol awakening response. AUC: area under the diurnal cortisol curve. EDSlope: early decline slope. ODSlope: overall decline slope. LDSlope: Late decline slope. MultiPhen: MultiPhen test [18] combining the seven cortisol features. EUR: European Americans. AFA: African Americans. HIS: Hispanic Americans. Meta-WZ [36]: meta-analysis using weighted Z-score test. Meta-F [37]: meta-analysis using Fisher’s probability test. Meta-WF: meta-analysis using weighted Fisher’s probability test. Age, gender and top five principal components were adjusted as covariates. Each cell presents the p-value. P-values less 0.05 are bolded. SNP-level Bonferroni threshold = 1.4×10^−5^ for single cortisol analysis, 0.0001 for MultiPhen.

## Discussion

This candidate gene association study investigated the associations between a’priori selected stress response genes and cortisol features in a multi-ethnic population by utilizing a gene-level multi-phenotype analysis approach. We found statistical evidence that variation in established stress response gene regions is related to features of the diurnal cortisol curve. One of the six stress response gene regions, ADRB2 had a significant association in a joint meta-analysis of multiple cortisol features. There were significant associations in European Americans, suggestive associations in African Americans, but no associations in Hispanic Americans. This gene encodes beta-2-adrenergic receptor which is in the G protein-coupled receptor superfamily [39]. It plays a role in blood pressure regulation, and influences both resting and stress-related blood pressure [15].

The SKAT methodology used for this gene-level analysis has several advantages as a set-based variance component test. First, SKAT is a powerful method that can integrate all SNPs in the region and can reduce the test degree of freedom when the SNPs are correlated due to linkage disequilibrium, which is of particular importance given the small ethnic group specific sample sizes for these analyses. Second, SKAT allows for the individual variant effects to vary from mean zero in either direction, and does not lose power when variants have different direction of effect unlike the Cohort Allelic Sum Test (CAST) [40] or Weighted Sum Statistic (WSS) [41] which have optimal power properties when effects across the SNPs are in the same direction and are of similar magnitude. Thirdly, it allows for the adjustment of covariates and is computationally efficient. The multi-phenotype analysis proposed in this paper can further integrate the rich information contained in the seven cortisol features to give a more precise description of the diurnal cortisol curve and improve the power of identifying statistical associations. We note that this gene-level multi-phenotype analysis is especially powerful when the SNPs in the gene are correlated and there exists consistent associations between more than one phenotype and the gene.

The Fisher method is a simple and powerful approach to combine p-values to increase association signals. We used the Fisher method to combine gene based association test p-values across seven cortisol features and obtained more significant results than the individual phenotype tests. Permutation was used to obtain p-values of the Fisher method, which makes it computationally expensive. An alternative approach is to extend the SKAT method to multiple phenotypes tests [42]. We considered this multi-phenotype SKAT, however, we have found that power of this approach was very sensitive to underlying assumptions regarding homogeneity or heterogeneity of genetic effects across phenotypes. In contrast, the Fisher method provided robust power regardless of underlying genetic effects model.

The permutation procedure we used for the Fisher method can preserve the correlation among cortisol features and the correlation between cortisol features and confounders. This strategy has been used in genetics and other areas of biological sciences and provided a reasonable performance for type I error correction [43]. However, in presence of genotype-covariate association that is ignored in our permutation approach, we may incur slightly inflated Type 1 error as mentioned in Remark 2.

There are three main limitations to this work. The first is a design limitation due the use of HapMap imputed variants, which are not functional SNPs. However, as the HapMap tagging SNPs may be in linkage disequilibrium with causal SNPs they are still useful for identifying genomic regions of potential interest. Second, compliance with cortisol sampling protocols is necessary for estimating reliable cortisol features [44, 45]. Compliance with taking samples within 10 minutes the requested times was greatest for wakeup (68%) and bedtime (75%) collections, and poorest during the middle of the day, ranging from 43%-57%. Stability of the cortisol features is of particular importance for genetic analyses, compared to other MESA cortisol work, as the effect estimates of individual variants are expected to be modest and large variation in features estimates could mask true associations. A third limitation is that, in absence of other information about genes most relevant to HPA activity, we selected a set of candidate genes involved in stress responsivity generally. Several of these (including *ADRB2*) are more involved in sympathetic activity than HPA activity per se; however both systems are interrelated. In this sense our analyses serve to illustrate a useful methodologic approach to examine genetic predictors of cortisol than to identify the most relevant genes. Future analyses should include a broader set of potentially relevant genes.

Despite the limitations, this work is novel in the ability to integrate the information of multiple SNPs in the region, multiple cortisol features and multiple ethnic groups, which was possible through the use of the innovative SKAT and multi-phenotype methodologies as well as the unique, highly detailed cortisol phenotype information. The gene-level multi-phenotype approach allows us to address the concern that individual SNPs may not replicate across ethnic groups as well as cortisol features due to differences in underlying patterns of linkage disequilibrium, differences in allele frequencies [46, 47], and heterogeneous effects on different cortisol features. The multiple phenotype gene-based analyses presented here provide new insight into the relationship between stress response genes and cortisol features and illustrate the advantage of integrating information in both the phenotype and genotype space by considering different hypotheses regarding marginal or joint association between a set of cortisol features and a set of SNPs, rather than analyzing each SNP and each cortisol feature in isolation. The multivariate association analysis at a gene level can enhance power of gene discovery tests by considering the correlation structure of the SNPs and the correlation structure between the multiple cortisol features.

## Supporting Information

S1 Fig LocusZoom plot of the association between all SNPs in the *ADRB2* gene region and cortisol features in European Americans. Each SNP in the *ADRB2* gene region was analyzed by MultiPhen (O’Reilly et al., 2012). The p-values were plotted using LocusZoom (Pruim et al., 2010).
(TIFF)

S2 Fig LocusZoom plot of the association between all SNPs in the *ADRB2* gene region and cortisol features in African Americans. Each SNP in the *ADRB2* gene region was analyzed by MultiPhen (O’Reilly et al., 2012). The p-values were plotted using LocusZoom (Pruim et al., 2010).
(TIFF)

S3 Fig LocusZoom plot of the association between all SNPs in the *ADRB2* gene region and cortisol features in Hispanic Americans. Each SNP in the *ADRB2* gene region was analyzed by MultiPhen (O’Reilly et al., 2012). The p-values were plotted using LocusZoom (Pruim et al., 2010).
(TIFF)

S1 Tables Supplementary Tables A—D. (DOCX)
