# Supplementary figures and images for: Association between Stress Response Genes and Features of Diurnal Cortisol Curves in the Multi-Ethnic Study of Atherosclerosis: A New Multi-Phenotype Approach for Gene-Based Association Tests
Source: PLoS One. 2015 May 20;10(5):e0126637. doi: 10.1371/journal.pone.0126637 (PMC4439141; doi:10.1371/journal.pone.0126637)

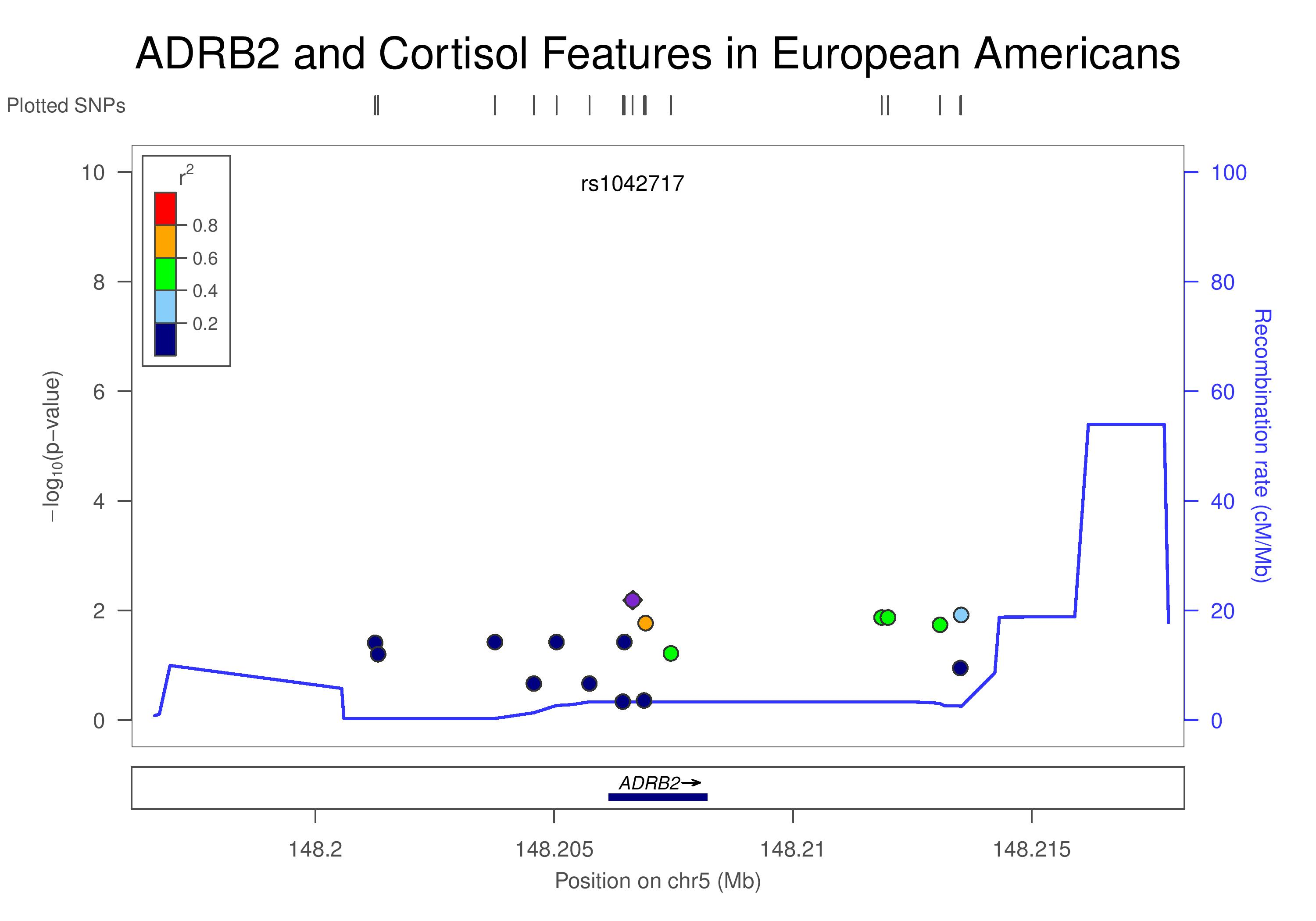

Supplement: S1 Fig — Each SNP in the ADRB2 gene region was analyzed by MultiPhen (O’Reilly et al., 2012). The p-values were plotted using LocusZoom (Pruim et al., 2010). (TIFF) [file pone.0126637.s001.tiff]

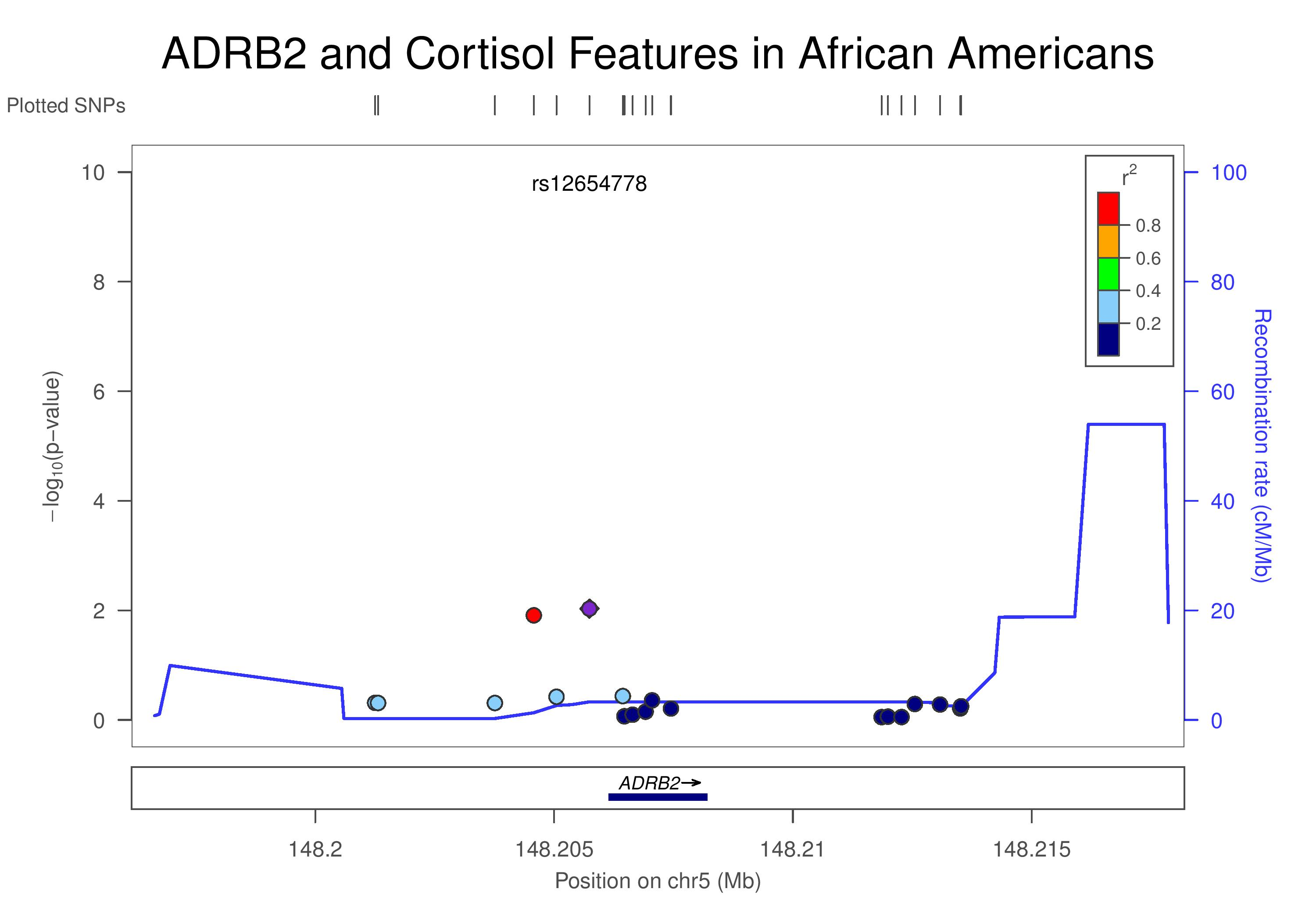

Supplement: S2 Fig — Each SNP in the ADRB2 gene region was analyzed by MultiPhen (O’Reilly et al., 2012). The p-values were plotted using LocusZoom (Pruim et al., 2010). (TIFF) [file pone.0126637.s002.tiff]

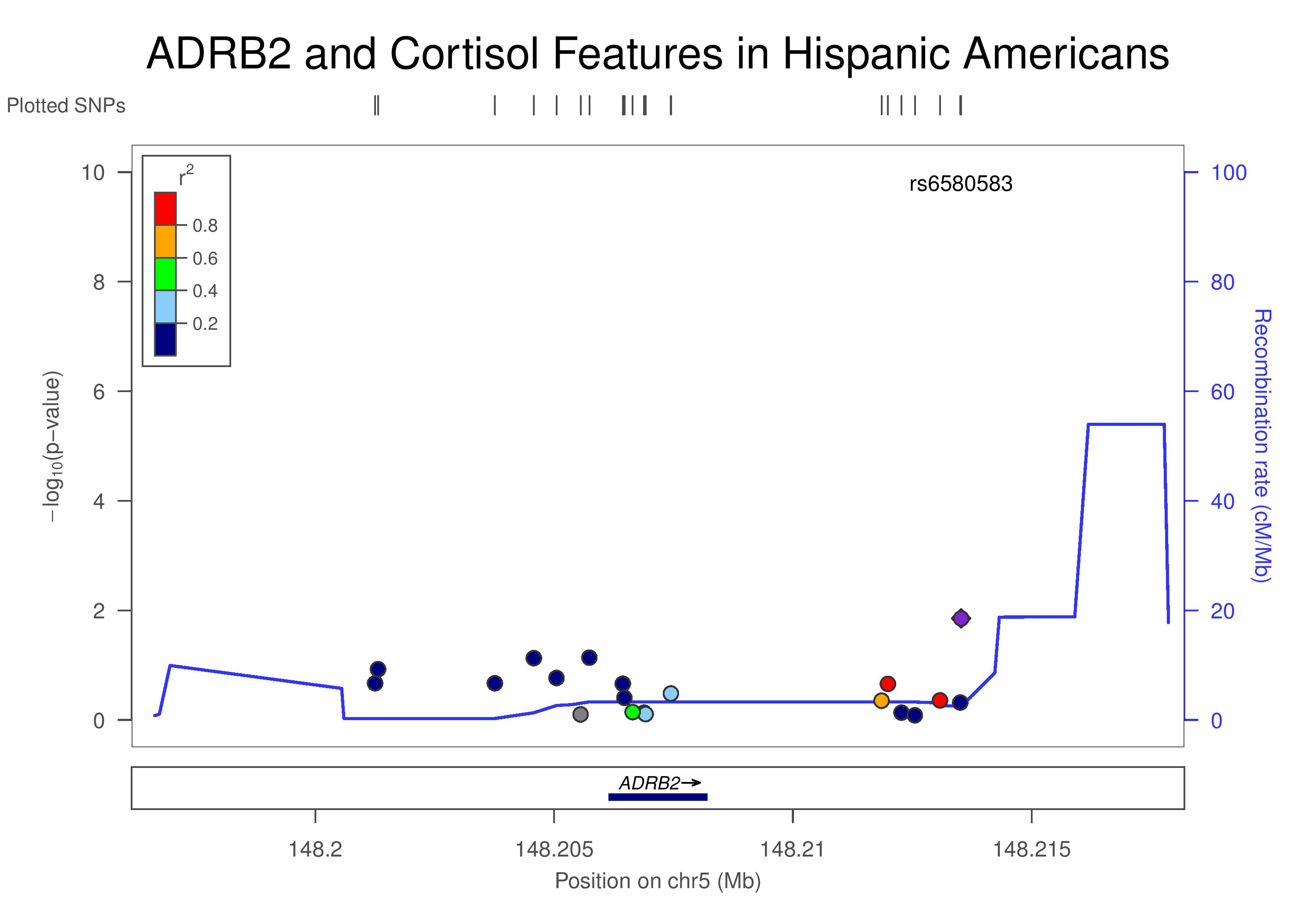

Supplement: S3 Fig — Each SNP in the ADRB2 gene region was analyzed by MultiPhen (O’Reilly et al., 2012). The p-values were plotted using LocusZoom (Pruim et al., 2010). (TIFF) [file pone.0126637.s003.tiff]
