# Supplementary material for: Association between Stress Response Genes and Features of Diurnal Cortisol Curves in the Multi-Ethnic Study of Atherosclerosis: A New Multi-Phenotype Approach for Gene-Based Association Tests
Source: PLoS One. 2015 May 20;10(5):e0126637. doi: 10.1371/journal.pone.0126637 (PMC4439141; doi:10.1371/journal.pone.0126637)
Supplement: S1 Tables — (DOCX) [file pone.0126637.s004.docx]

**Supplementary Tables**

**Table A. Cortisol features of the diurnal curve.**

| **Cortisol Feature** | **Abbreviation** | **Description** |
| --- | --- | --- |
| Wakeup | Wakeup | Average cortisol concentration from wakeup for an individual |
| Cortisol awakening response | CAR | The average difference in cortisol concentrations between the peak and wakeup measurements |
| Bedtime | Bedtime | Average cortisol concentration at bedtime for an individual |
| Area under the curve | AUC | Standardized AUC for the interval 0hr-16hr since wakeup averaged across all days for an individual |
| Early Decline Slope | EDSlope | The slope from 0.5 hours and 2 hours since wakeup pooled across all days for an individual |
| Overall Decline Slope | ODSlope | The overall decline slope ignoring the peak value from wakeup to bedtime pooled across all days for an individual |
| Late Decline Slope | LDSlope | The slope from 2 hours to 16 hours since wakeup pooled across all days for an individual |

**Table B. Pearson correlation coefficients between cortisol features.**

|  |  | **Wakeup** | **CAR** | **Bedtime** | **AUC** | **EDSlope** | **ODSlope** | **LDSlope** |
| --- | --- | --- | --- | --- | --- | --- | --- | --- |
| EUR | Wakeup | 1.00 |  |  |  |  |  |  |
|  | CAR | **-0.51** | 1.00 |  |  |  |  |  |
|  | Bedtime | **0.25** | -0.08 | 1.00 |  |  |  |  |
|  | AUC | **0.42** | 0.05 | **0.66** | 1.00 |  |  |  |
|  | EDSlope | **-0.24** | **-0.26** | 0.03 | **0.15** | 1.00 |  |  |
|  | ODSlope | **-0.40** | **0.29** | **0.30** | **0.19** | 0.04 | 1.00 |  |
|  | LDSlope | -0.04 | -0.04 | **0.37** | **0.28** | **-0.36** | **0.77** | 1.00 |
| AFA | Wakeup | 1.00 |  |  |  |  |  |  |
|  | CAR | **-0.51** | 1.00 |  |  |  |  |  |
|  | Bedtime | **0.25** | -0.08 | 1.00 |  |  |  |  |
|  | AUC | **0.42** | 0.05 | **0.66** | 1.00 |  |  |  |
|  | EDSlope | **-0.24** | **-0.26** | 0.03 | **0.15** | 1.00 |  |  |
|  | ODSlope | **-0.40** | **0.29** | **0.30** | **0.19** | 0.04 | 1.00 |  |
|  | LDSlope | -0.04 | -0.04 | **0.37** | **0.28** | **-0.36** | **0.77** | 1.00 |
| HIS | Wakeup | 1.00 |  |  |  |  |  |  |
|  | CAR | **-0.43** | 1.00 |  |  |  |  |  |
|  | Bedtime | **0.41** | **-0.15** | 1.00 |  |  |  |  |
|  | AUC | **0.52** | 0.03 | **0.76** | 1.00 |  |  |  |
|  | EDSlope | **-0.22** | **-0.29** | -0.03 | 0.07 | 1.00 |  |  |
|  | ODSlope | **-0.33** | **0.21** | **0.47** | **0.43** | -0.02 | 1.00 |  |
|  | LDSlope | 0.07 | **-0.09** | **0.52** | **0.32** | **-0.42** | **0.73** | 1.00 |
| ALL | Wakeup | 1.00 |  |  |  |  |  |  |
|  | CAR | **-0.37** | 1.00 |  |  |  |  |  |
|  | Bedtime | **0.38** | **-0.12** | 1.00 |  |  |  |  |
|  | AUC | **0.51** | 0.03 | **0.72** | 1.00 |  |  |  |
|  | EDSlope | **-0.28** | **-0.30** | -0.06 | 0.04 | 1.00 |  |  |
|  | ODSlope | **-0.35** | **0.17** | **0.42** | **0.36** | 0.05 | 1.00 |  |
|  | LDSlope | **0.08** | **-0.08** | **0.50** | **0.32** | **-0.39** | **0.70** | 1.00 |

*CAR: cortisol awakening response. AUC: area under the diurnal cortisol curve. EDSlope: early decline slope. ODSlope: overall decline slope. LDSlope: Late decline slope. EUR: European Americans. AFA: African Americans. HIS: Hispanic Americans. Significant correlation coefficients (p-value < 0.05) are bolded.*

**Table C. Univariate association analysis between covariates and cortisol features by ANOVA.**

|  |  | **Wakeup** | **CAR** | **Bedtime** | **AUC** | **EDSlope** | **ODSlope** | **LDSlope** |
| --- | --- | --- | --- | --- | --- | --- | --- | --- |
| EUR | Age | 0.6497 | 0.1574 | **0.0002** | 0.1586 | **0.0081** | 0.3133 | 0.8468 |
|  | Gender | 0.8432 | 0.3854 | 0.8473 | **0.0399** | **0.0252** | 0.0746 | 0.2781 |
|  | Site | 0.4611 | 0.4203 | 0.7845 | 0.6579 | 0.6479 | 0.9093 | 0.3230 |
|  | Education | 0.3201 | 0.5873 | 0.7661 | 0.3445 | 0.1794 | 0.6262 | 0.8626 |
| AFA | Age | 0.0643 | 0.4062 | 0.0513 | **0.0142** | 0.5508 | 0.6638 | 0.1717 |
|  | Gender | 0.7587 | 0.1099 | 0.8023 | 0.1844 | **0.0009** | 0.5168 | 0.4500 |
|  | Site | 0.9748 | 0.9215 | 0.4192 | 0.4094 | 0.0755 | 0.3726 | 0.1882 |
|  | Education | 0.1289 | **0.0443** | 0.9207 | 0.4755 | 0.7824 | 0.5258 | 0.4160 |
| HIS | Age | **0.0014** | 0.1525 | **6.48E-13** | **1.20E-09** | 0.1636 | **0.0003** | **0.0086** |
|  | Gender | 0.2637 | 0.6040 | 0.7223 | **0.0011** | **0.0490** | 0.1959 | 0.6427 |
|  | Site | **0.0057** | 0.1106 | 0.2982 | 0.1070 | 0.1120 | **0.0007** | 0.3606 |
|  | Education | 0.6906 | 0.4700 | 0.2281 | 0.6049 | 0.2477 | 0.1405 | 0.3710 |
| ALL | Age | 0.6278 | 0.9857 | **1.88E-06** | **1.97E-06** | 0.7136 | 0.5142 | 0.6990 |
|  | Gender | 0.2901 | 0.1383 | 0.8118 | **0.0001** | **4.70E-05** | **0.0434** | 0.6953 |
|  | Site | 0.1602 | 0.4864 | 0.0520 | **0.0088** | 0.1283 | **0.0061** | 0.1765 |
|  | Education | 0.3309 | 0.0693 | 0.7863 | 0.6466 | 0.1305 | 0.4651 | 0.3956 |
|  | Race | **3.59E-05** | 0.1122 | **3.78E-13** | **5.13E-06** | **0.0014** | **0.0001** | **1.95E-06** |

*CAR: cortisol awakening response. AUC: area under the diurnal cortisol curve. EDSlope: early decline slope. ODSlope: overall decline slope. LDSlope: Late decline slope. EUR: European Americans. AFA: African Americans. HIS: Hispanic Americans. P-values less 0.05 are bolded.*

**Table D. Most significant SNPs in the six stress response genes obtained by single SNP meta analysis of the association between the cortisol features and genetic variants (Method 1). The last column is a result of Method 2 where each SNP is tested with multiple cortisol features jointly.**

|  |  | **Wakeup** | **CAR** | **Bedtime** | **AUC** | **EDSlope** | **ODSlope** | **LDSlope** | **MultiPhen** |
| --- | --- | --- | --- | --- | --- | --- | --- | --- | --- |
| ADRA2A | Top SNP | rs12246561 | rs11195417 | rs11815669 | rs11195419 | rs11195418 | rs11195419 | rs11195419 | rs11195418 |
|  | P-value | **0.0177** | 0.0652 | 0.0658 | **0.0300** | **0.0016** | 0.0753 | 0.1843 | **0.0037** |
| ADRB2 | Top SNP | rs11168066 | rs1432622 | rs3857420 | rs6580583 | rs6580583 | rs12654778 | rs1042719 | rs6580583 |
|  | P-value | **0.0376** | **0.0042** | **0.0043** | **0.0188** | **0.0422** | **0.0005** | **0.0115** | **0.0051** |
| NR3C1 | Top SNP | rs258750 | rs10477211 | rs10482668 | rs33389 | rs10482668 | rs12521436 | rs10482682 | rs10482616 |
|  | P-value | **0.0219** | 0.0510 | **0.0224** | **0.0085** | 0.1033 | **0.0211** | 0.0562 | 0.1137 |
| NR3C2 | Top SNP | rs4835490 | rs6847959 | rs11732130 | rs6834935 | rs17024443 | rs11099685 | rs11099685 | rs5534 |
|  | P-value | **0.0140** | **0.0052** | **0.0021** | **0.0237** | **0.0310** | **0.0056** | **0.0046** | **0.0008** |
| SLC6A4 | Top SNP | rs16965581 | rs3794808 | rs9903602 | rs2020941 | rs2020942 | rs16965623 | rs16965623 | rs16965581 |
|  | P-value | 0.1316 | 0.0940 | 0.2302 | **0.0143** | **0.0392** | **0.0248** | **0.0330** | **0.0087** |
| TH | Top SNP | rs10743149 | rs10770140 | rs4930046 | rs4930046 | rs10743149 | rs4930046 | rs10840490 | rs10770141 |
|  | P-value | 0.0700 | **0.0364** | **0.0222** | **0.0115** | **0.0344** | **0.0258** | **0.0075** | **0.0062** |

*CAR: cortisol awakening response. AUC: area under the diurnal cortisol curve. EDSlope: early decline slope. ODSlope: overall decline slope. LDSlope: Late decline slope. MultiPhen: the MultiPhen method combining seven cortisol features. The Fisher’s probability test combining the three ethnicities is used for this meta analysis. Age, gender and top five principal components were adjusted as covariates. P-values less 0.05 are bolded. SNP-level Bonferroni threshold = for single cortisol analysis..*
